# Epidemiology of Musculoskeletal Injuries Among Climbers—A Systematic Review

**DOI:** 10.3390/jfmk11010019

**Published:** 2025-12-30

**Authors:** Jakub Zieliński, Monika Grygorowicz, Jacek Lewandowski

**Affiliations:** 1 Department of Musculoskeletal Physiotherapy, Poznan University of Physical Education, 61-871 Poznan, Poland; 2 Departament of Physiotherapy, Poznan University of Medical Sciences, 60-512 Poznan, Poland; 3 Department of Physical Therapy and Wellness, Calisia University, 62-821 Kalisz, Poland; lewandowski@awf.poznan.pl

**Keywords:** sport climbing, injury risk factors, preventive measures

## Abstract

Lead climbing and bouldering have witnessed a surge in popularity, particularly highlighted by their inclusion in prestigious events like the 2020 Summer Olympic Games in Tokyo. This systematic review aims to comprehensively assess existing literature on injury risk factors and prevention programs specific to these disciplines. We systematically searched PubMed, Web of Science, and SPORTDiscus up to November 2023. Methodological quality was appraised using the Joanna Briggs Institute (JBI) critical appraisal tools. Data synthesis involved qualitative analysis. Of 463 screened records, 7 studies were included, encompassing data from over 4000 climbers. The literature consistently indicates that overuse injuries—particularly to the fingers and shoulders—are more prevalent than acute injuries in adult population. However, evidence for specific risk factors is inconclusive and contradictory. Reported associations for higher skill level, age, and use of preventive measures (e.g., taping) were inconsistent across studies. Further research employing rigorous methodologies and long-term follow-up is warranted to elucidate injury mechanisms in lead climbing and bouldering. These investigations are crucial for informing clinical practice and developing sport-specific injury prevention strategies aimed at ensuring the safety and well-being of athletes in these disciplines. Future studies should focus on standardizing injury definitions and assessment methods and explore targeted preventive measures to address the unique risks associated with these sports.

## 1. Introduction

Lead climbing and bouldering have witnessed a surge in popularity, not only as competitive activities but also as accessible pursuits for individuals spanning various age groups. In recent years, in the USA alone, over 9 million people have actively participated in climbing, which has gained recognition by being included in prestigious sporting events such as the 2020 Summer Olympic Games in Tokyo. This acknowledgement reflects the global upsurge in interest and participation in a sport known for its health benefits and historically low injury rates [1,2].

Nonetheless, the landscape of modern climbing, along with evolving training methodologies [3], has ushered in a new spectrum of climbing-related injuries. Previous studies have primarily focused on injuries related to flexor tendon pulleys [4,5]. Climbing is indeed a sport that places substantial demands on the fingers [6] and the musculoskeletal system as a whole [7]. However, the risk factors associated with rock climbing injuries remain largely unexplored, despite the growing interest and engagement in this demographic [8].

The prevailing consensus highlights potential risk factors, including age, years of climbing experience, the highest climbing grade achieved (skill level), a high climbing intensity score (CIS), and participation in lead climbing [9]. Recently, a systematic review was published, highlighting numerous risk factors that appear to increase the likelihood of overuse injuries among climbers, thus emphasizing the challenge posed by the low quality of included papers [10]. Notably, the quality assessment in this instance was conducted using the Downs and Black Quality Index, a prevalent metric designed specifically for evaluating the quality of clinical trial methodologies [11,12]. Nevertheless, the reliance on the Downs and Black instrument warrants scrutiny, given its origin in clinical trial contexts, potentially misaligning with the diverse methodological frameworks embraced across the spectrum of studies encompassed within the systematic review. A lot of publications exploring injury epidemiology within the sporting populace employ cross-sectional study designs [10,13,14,15]. These studies deviate from clinical trials in their methodological orientation and investigative aims, thereby raising doubts about the suitability of the Downs and Black tool for comprehensive quality evaluation. Acknowledging this possible limitation, the authors of the current systematic review applied an alternative quality assessment methodology. The Joanna Briggs Institute (JBI) quality evaluation framework was chosen, meticulously tailored to accommodate the nuanced methodological paradigms inherent in various publication types, including cross-sectional studies [16,17,18,19,20].

Thus, given the backdrop of climbing’s expanding popularity and the expected increase in older climbing enthusiasts, this study aims to investigate injury risk factors specific to lead climbing and bouldering using JBI quality assessment tools. By reviewing scientific articles focused on lead climbing injuries and associated risk factors, this research seeks to shed light on the complex landscape of injuries among lead climbing athletes, providing essential insights into injury prevention and management within this growing demographic.

## 2. Materials and Methods

### 2.1. Data Collection Process and Search Strategy

The study conducted searches across the following databases: PubMed, Web of Science, and SportDiscuss. The search incorporated keywords including (mountaineering OR climb* OR boulder*) AND (wounds and injuries OR athletic injuries OR sport injury) AND risk factors AND (prevention OR intervention OR safety). It is noteworthy that this search strategy had been previously employed in a systematic review by Woollings et al. in 2015 [9]. In the present study, this strategy was employed to determine whether any new original research within this domain had been published since then. The literature search was initially performed on 14 February 2023, and subsequently updated on 24 November 2023.

### 2.2. Eligibility Criteria

Inclusion Criteria: The inclusion criteria encompassed studies grounded in original data, conducted on lead climbers or boulderers. The outcomes of interest included injuries related to lead climbing, along with one or more potential risk factors or prevention strategies. Eligible study designs comprised randomized controlled trials, quasi-experimental studies, cohorts, cross-sectional investigations, case–control studies, longitudinal studies, and case series studies. The studies had to be peer-reviewed and published in the English language. Conversely, review articles and case studies were excluded from consideration.

### 2.3. Quality Assessment

To assess the quality of the selected studies, the JBI Critical Appraisal Checklist for analytical cross-sectional studies, designed to evaluate studies that analyze data collected at a single point in time [21], and the JBI Critical Appraisal Checklist for cohort studies [22] were utilized applying relevant checklists [23,24]. The JBI Critical Appraisal Checklists were used to ensure a rigorous and systematic evaluation of the included studies. These checklists offer a standardized method for assessing study quality, addressing aspects such as study design validity, data collection accuracy, and statistical analysis appropriateness. Their use enhances the consistency, transparency, and reliability of the review process, ensuring valid conclusions [16,17,18,19,20]. Each author conducted independent assessments, and subsequently, the results were compared, and agreement was achieved through in-depth face-to-face discussion between two raters.

## 3. Results

**Figure 1:**
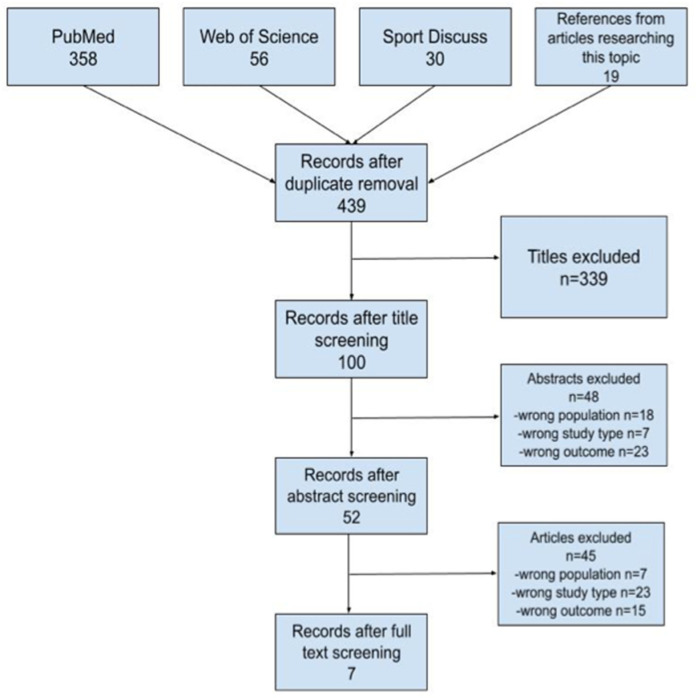
Flowchart of selected papers.

The initial literature search yielded a total of 463 potentially relevant articles. Subsequently, after reviewing the titles, 439 articles were chosen for abstract screening. Following a detailed examination, 52 articles were subjected to full-text evaluation. Ultimately, seven articles were identified as meeting the predefined inclusion criteria (Figure 1).

A total of 1401 climbers were included in the analysis from studies [9,10,11,12,13,14,15]. The age range of participants varied from adolescents [25] to older athletes [26]. Study characteristics included in this systematic review are presented in Table 1. The study by Pirruccio et al. [27] analyzed 2292 unique, unweighted cases but did not provide data about the number of participants involved.

**Figure 2:**
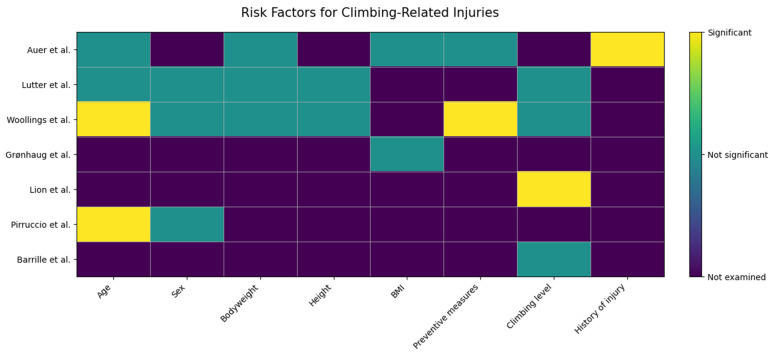
Heatmap of Climbing Study Risk Factors. Auer: [25], Lutter: [29], Woollings: [26], Grønhaug: [27], Lion: [28], Pirruccio: [31], Barrile: [30].

In addition to presenting the data in the form of traditional paragraphs detailing individual results, we also opted for a more visual communication of complex data. In the heatmap below (Figure 2), the rows represent the different studies, while the columns represent the elements or risk factors assessed. Green indicates a significant positive impact, red indicates a significant negative impact, gray indicates an insignificant impact, and a cross indicates that the factor was not tested in the study in question.

### 3.1. Injury Definition and Type

The reviewed studies lacked a consistent injury classification; however, the two that differentiated mechanisms provide critical insight into distinct injury etiologies. The apparent contradiction between studies—with Lutter et al. [29] finding 68% overuse injuries and Barille et al. [26] finding 61% acute—likely reflects differing study populations or settings (e.g., elite vs. recreational climbers, training vs. competition). This divergence underscores that injury patterns are context-dependent, but crucially, both studies confirm that overuse/chronic injuries constitute a substantial proportion (39–68%) of the total injury burden. Therefore, injury prevention cannot focus solely on acute trauma but must also address the cumulative load inherent to climbing training.

### 3.2. Measures of Injury

In terms of injury incidence, two studies provided rates per 1000 h of climbing: Woollings et al. reported an incidence of 4.44/1000 h [30], and Auer et al. stated an incidence of 2.7/1000 h. The remaining studies reported the absolute number of injuries: 305 in Auer et al. [28], 275 in Lutter et al. [29], 385 in Grønhaug et al. [25], 356 in Lion et al. [31], and 2292 in Barille et al. [26].

### 3.3. Risk Factors

The seven research projects [25,26,27,28,29,30,31] collectively presented 35 potential factors that could influence the occurrence of climbing injuries, with 26 of these factors being individually investigated in separate studies.

### 3.4. Age

The role of age as a risk factor presents a complex and non-linear relationship. While four studies investigated its influence, the findings were contradictory, likely reflecting differences in study populations. Research focusing on adult cohorts found no significant association between age and injury risk [26,28,29]. In contrast, a study on youth climbers identified a critical period, reporting that adolescents aged 15–19 had an 11.3 times higher injury risk than those aged 11–14 [30]. This suggests that age is not a simple linear risk factor but may be most significant during phases of rapid growth and increased training intensity in youth athletes, while its effect plateaus in adulthood.

### 3.5. Climbing Level

Evidence regarding climbing level and injury risk points toward a potential threshold effect. Although three studies found no significant association [25,26,30], one key study by Lion et al. demonstrated a clear dose–response relationship: injury odds increased with climbing grade, from 3.95 for levels 6b–6c+ to 6.05 for levels ≥7b+ [31]. This indicates that advancing to higher climbing levels (approximately ≥6b) may be a significant risk factor, potentially due to increased mechanical stress, more intense training, and greater exposure to difficult moves. The conflicting results may stem from variations in sample composition, with studies including more beginner/intermediate climbers diluting the effect seen at elite performance levels.

### 3.6. Sex

The analyzed literature uniformly refutes bodyweight as a direct, independent risk factor for climbing injuries. All three studies investigating this variable, across adult and youth populations, found no significant association between a climber’s weight and the incidence of either acute or overuse injuries [28,29,30]. This consistent null finding suggests that injury etiology in climbing is more strongly influenced by other factors such as load management, technique flaws, or muscle-tendon imbalances, rather than absolute body mass. The sport’s emphasis on strength-to-weight ratio may indirectly manage this variable within the climbing population.

### 3.7. Bodyweight

The evidence uniformly indicates that absolute bodyweight is not an independent risk factor for injury in climbing. All three studies investigating this variable—spanning adult [28,29] and youth [30] populations—found no significant association with injury incidence. Auer et al., who examined 507 climbers aged 30 ± 8, reported that there was no significant difference in body weight (*p* = 0.85; OR 1.01; 95% CI 0.99–1.02) between participants affected by injuries and those who were not. Lutter et al., in their study of 198 climbers aged 35 and over, found no significant association between a climber’s weight and the development of acute or overuse injuries [28]. Woollings et al., who focused on youth sport climbers, found no significant association between weight and the development of injuries [30]. This consistent null finding suggests that injury mechanics are likely governed by relative load (strength-to-weight ratio), technique, or training errors rather than by body mass alone.

### 3.8. Height

The available data do not support a relationship between a climber’s height and injury risk. Both studies that examined this anthropometric factor, in adult [29] and youth [30] cohorts, found no statistically significant association. Given this limited but unanimous evidence, height does not appear to be a primary or modifiable risk factor for climbing-related injuries. Neither Lutter et al., who examined 198 climbers aged 35 and over, nor Woollings et al., who studied 116 climbers aged 11–19, found any statistically significant differences between these variables [29,30].

### 3.9. BMI

Similarly to findings on bodyweight and height, BMI does not emerge as a significant predictor of injury risk in this review. Two studies assessing BMI in adult climbers found no meaningful association with injury occurrence [25,28]. Auer et al., who examined 507 climbers aged 30 ± 8, found no meaningful difference in body mass index (BMI) (*p* = 0.88; OR 1.01; 95% CI 0.93–1.08) among individuals affected by injuries compared to those who were not [28]. Grønhaug, who studied 667 climbers, also found no association between climbing-related chronic injuries and BMI [25]. This further reinforces the conclusion that general anthropometric indices are less relevant to injury etiology in climbing than sport-specific factors like load management and movement patterns.

### 3.10. Preventive Measures

The relationship between preventive taping and injury risk is counterintuitive and warrants careful interpretation. Two studies, both in youth populations, reported that finger taping was associated with a significantly higher odds of injury (OR 5.09 [30] and 1.4 [26]). A third study in adults found no protective effect [28]. Importantly, this association likely reflects confounding by indication, where climbers at higher risk (e.g., those with prior pain or higher training loads) are more likely to use tape. Woollings et al. reported, after analyzing 116 climbers aged 11–19, a statistically significant difference between the use of preventive measures and injury occurrence. The odds ratio (OR) for sustaining an injury among individuals who used preventive taping was 5.09 times higher (95% CI 1.44 to 18.02) compared to those who did not use it [30]. Barrile et al., in their study of 52 climbers aged 7–18, found that taping fingers was associated with an increased odds of reported injury by a factor of 1.4 (95% CI 1.4–4.5, *p* = 0.04) when adjusted for climbing hours [26]. Auer et al. concluded that preventive measures did not demonstrate a protective effect against the occurrence of injuries based on a sample of 507 climbers aged 30 ± 8 [28]. Therefore, tape use should be interpreted as a marker of elevated risk rather than a causative factor.

### 3.11. Activity in Other Climbing Sports

Participation in other sports was investigated as a potential risk factor in two studies [26,28], with neither finding a statistically significant association. Based on current evidence, cross-sport activity does not appear to measurably alter climbing injury risk.

### 3.12. Other Risk Factors

Several distinct risk factors were individually investigated in separate studies. A wide array of additional factors—including warm-up routines, prior injury, equipment use (e.g., fingerboards, shoe characteristics), training habits, and socioeconomic status—were each examined in single studies. While this highlights the multifactorial nature of injury risk, the lack of replicated investigation for these variables precludes any definitive conclusion regarding their significance. They represent important avenues for future, targeted research.

### 3.13. Methodological Assessment

The methodological quality of the seven included studies was acceptable, with all meeting the threshold for inclusion. Six cross-sectional studies [25,26,27,29,30,31] satisfied key criteria regarding sampling and measurement (Table 2). The sole prospective cohort study [28] scored 6 out of 11 on its checklist. A key limitation across the literature is the predominance of cross-sectional designs, which inherently limits the ability to establish temporal causality between risk factors and injuries.

### 3.14. Synthesis of Risk Factors

The most consistent findings highlight intrinsic sport demands and specific high-risk groups. The predominance of overuse mechanisms, accounting for 39–68% of injuries [26,29], establishes repetitive microtrauma as the foundational injury etiology. Furthermore, high climbing proficiency (grades ≥ 6b) was identified as a significant, dose-dependent risk factor, with one study demonstrating odds ratios increasing from 3.95 to 6.05 with higher grades [31]. Adolescent age (15–19 years) was also a significant risk factor in one study (OR 11.3) [30], indicating a critical period of vulnerability likely related to growth and training intensity.4.5.2.

Several demographic and anthropometric variables were consistently not associated with injury risk. Biological sex showed no significant association across studies [26,29,31]. Similarly, bodyweight, height, and Body Mass Index (BMI) were uniformly found to be non-significant [25,28,29,30], suggesting injury risk is independent of these basic physical characteristics in the studied cohorts.

Notable findings require careful interpretation. The association between preventive finger taping and higher injury odds [26,30] is likely due to confounding by indication, where taping is a marker of pre-existing pain or high-risk behavior rather than a cause. Finally, a wide array of other factors (e.g., warm-up routines, prior injury) were examined in single studies only. This, combined with the predominantly cross-sectional design of the evidence, precludes definitive causal conclusions for many potential risks and highlights a need for longitudinal, multi-variable research.

## 4. Discussion

In this review, our objective was to explore the landscape of rock-climbing injuries and identify potential risk factors associated with the sport. The consensus among existing literature has emphasized potential risk factors, including age, climbing experience, skill level, climbing intensity, and participation in lead climbing [6]. However, it is crucial to acknowledge the variability in injury definitions, study populations, and methodological quality across the reviewed studies. This divergence contributes to a notable range in reported injury rates and introduces challenges in drawing definitive conclusions regarding risk factors. Despite these variations, it is essential to highlight that certain modifiable potential risk factors have surfaced, holding significance for future interventions aimed at injury prevention in the realm of lead climbing [25,26,27,28,29,30,31].

This study conducted a comprehensive exploration of lead climbing injuries and their associated risk factors to shed light on the nuanced nature of injuries among athletes engaged in lead climbing and bouldering. Through a systematic review of scientific articles dedicated to this subject, this research aimed to provide crucial insights into injury prevention and management within this growing demographic. The synthesis of the identified studies outlined various risk factors associated with climbing injuries. Factors such as age, climbing level, sex, body weight, height, BMI, and preventive measures were examined across different studies. However, the examination of these factors yielded disparate results.

For instance, while some studies identified age [27,30] and climbing level [31] as potential risk factors, others found no statistically significant associations [26,28,29,30]. According to research, younger climbers may have less physical conditioning and experience, leading to a higher risk of injuries due to improper techniques or overestimating their capabilities. Moreover, younger athletes often lack the physical maturity and conditioning necessary to avoid injuries, and their inexperience can result in the use of inefficient or improper techniques, increasing their risk of falls and overuse injuries [2]. Additionally, the enthusiasm and confidence of younger climbers can lead them to overestimate their abilities, further contributing to the likelihood of injury [10]. On the other hand, older climbers may face an increased risk of injury due to the natural decline in physical strength, flexibility, and recovery ability associated with aging. This decline makes them more susceptible to overuse injuries and strains. Research indicates that as individuals age, there is a significant reduction in muscle force and power, balance, and reaction times, which contributes to an elevated risk of injuries, particularly in activities that require sustained physical effort like climbing [32,33]. Additionally, age-related decreases in flexibility and slower recovery rates further heighten the vulnerability of older climbers to strains and overuse injuries [10].

Beginner climbers are more likely to use inefficient or improper techniques, which increases the risk of falls and overuse injuries. This is because they often lack the experience and technical skill necessary to climb safely, leading to improper body positioning and movements that can result in injuries such as tendonitis and muscle strains. This is primarily because repetitive movements and poor technique place unnecessary strain on specific muscles and joints, increasing the risk of injuries such as tendinitis and stress fractures. Without proper form, certain muscles and joints are overloaded, leading to microtrauma that, if not given adequate time to heal, can accumulate into more severe injuries. Conversely, advanced climbers may push their limits more aggressively, taking on more challenging routes that carry a higher risk of injury. This includes more complex moves that put additional stress on the fingers, elbows, and shoulders, often leading to overuse injuries and acute trauma from falls or slips during high-difficulty climbs [10]. Less experienced climbers might not recognize the early signs of overtraining. Inadequate rest and recovery periods do not allow the body to repair these micro-injuries, further exacerbating the risk of chronic injury. On the other hand, more experienced climbers might take calculated risks that could still lead to injuries, especially if they underestimate their fatigue or overestimate their abilities [34].

Similarly, variables like sex, body weight, height, and BMI showed no meaningful influence on injury occurrence, according to the collective findings of the reviewed studies [25,26,27,28,29,30,31]. The assessment of preventive measures also produced conflicting results. Barrile et al. found that toe taping was associated with an increased risk of injury and speculated that climbers use taping to return to activity prematurely, leading to a higher risk of chronic injury [26]. Along the same lines, Woollings et al. also speculated that individuals who use preventive taping might lower their vigilance and rationality, leading them to engage in more aggressive and risky climbing behavior, thereby increasing the risk of injury. In contrast Auer et al. found that use of the preventive taping did not reduce the injury rate [28]. Furthermore, the investigation of various other potential risk factors, ranging from warming up fingers and prior injury history [28] to engagement in other sports [26,28] and safety skills [26], revealed a wide array of factors that have been individually explored but lacked consistent associations across studies.

Understanding why such discrepancies exist is crucial for several reasons, since, in clinical decision-making, conflicting evidence can complicate the process, leading to uncertainty about the most appropriate interventions. Moreover, clear evidence on the effectiveness of preventive measures is essential for optimizing resource allocation in healthcare system. In the realm of injury prevention, compelling evidence supports the indispensability of strength training. Saeterbakken et al. investigated the interplay between resistance training, enhancement of climbing performance, and injury prevention. Their systematic review scrutinized existing literature to elucidate how the integration of resistance training can positively influence climbing performance while mitigating injury risks. Their findings advocate for the adoption of structured low-volume, high-resistance training regimens, such as biweekly sessions involving hanging from small ledges or a fingerboard, as a viable strategy for climbers [35]. Consequently, contemporary injury prevention protocols should prioritize augmenting performance levels by fostering resilience to loads pertinent to climbing activities, thus advocating for the widespread adoption of this approach within the climbing community. However, there is a limited number of studies covering adolescent climbers. It should be noted that primary periphyseal stress injuries are the most prevalent overuse injuries in this group, affecting the intricate epiphysial–physeal–metaphyseal complex; frequently mistaken for other finger injuries such as A2 ruptures, primary periphyseal stress injuries are often overlooked in skeletally immature climbers, highlighting the need for future studies to explore their pathophysiology, diagnosis, and management strategies [36].

It is important to acknowledge the limitations of this study. The variability in injury classifications across studies hindered the establishment of a unified understanding of injury incidence [25,26,27,28,29,30,31]. Additionally, the divergent findings across different risk factors highlight the complexity and variability in identifying definitive associations between these factors and climbing-related injuries. Variations in study design, sample size, and data collection methods can lead to differing results, as smaller sample sizes may lack the statistical power to detect significant associations. Differences in the demographic characteristics of study populations (e.g., age distribution, climbing experience, training habits) can affect the generalizability and consistency of findings. Moreover, since no scientifically thorough background exists for collecting epidemiological data for climbing, not every paper addresses relevant factors that might contribute to or affect the injury risk. This study underscores the necessity for further robust research to unravel the intricate web of risk factors contributing to climbing injuries. By addressing the limitations and inconsistencies observed in the current body of literature, future studies can refine our understanding and assist in the development of targeted injury prevention strategies for the expanding population engaged in sport climbing and bouldering.

## 5. Conclusions

This systematic review synthesizes the current, albeit heterogeneous, evidence on injuries in lead climbing and bouldering. The analysis reveals that overuse injuries constitute the dominant pattern in adult population, with one study reporting they comprise 68% of all injuries. Finger injuries, particularly to the pulley system, emerge as a frequent and early concern, highlighting a critical area for preventative focus. While reported injury incidence varies (2.7 to 4.44 per 1000 h), a notably elevated risk was identified for adolescent climbers (ages 15–19), with one study reporting an odds ratio of 11.3. For climbers and coaches, the most immediate, evidence-informed recommendation is to prioritize education and monitoring of training load to mitigate overuse and concerning finger stress. The effectiveness of specific interventions like taping remains unclear due to contradictory findings. The inconsistent results regarding risk factors such as skill level and age are largely attributable to methodological heterogeneity. Therefore, the primary imperative for future research is not merely more studies, but methodologically rigorous ones. Essential steps include standardizing injury definitions, employing prospective longitudinal designs with large samples, and implementing—and clearly reporting—blinded assessment and standardized intervention protocols. This approach is necessary to transform the current landscape of conflicting evidence into a reliable foundation for effective, evidence-based injury prevention strategies.

## Figures and Tables

**Table 1:** Study characteristics.

Primary Injury Outcome Measure (as Reported)	Injury Type	Population	Study Type	Aim	Study
305 injuries	NA	507 climbers, aged 30 ± 8	cohort	To prospectively evaluate the cause of injuries sustained during indoor bouldering, the proportion of affected body location, and injury severity and investigate the influence of potential preventive measures and risk factors on the injury rate	Auer, et al., 2021 [28]
275 injuries	acute 32%, overuse 68% (including degenerative—47%)	198 climbers, aged 35 and over	cross sectional	To analyze injury demographics, distribution, and severity for the older rock-climbing athlete.	Lutter et al., 2019 [29]
4.44/1000 climbing hours	NA	116 climbers, aged 11–19	cross sectional	To examine the incidence, mechanisms, and risk factors for injury in recreational and elite sport climbers and boulderers aged 11–19 years	Woollings et al., 2015 [30]
385 injuries	NA	667 climbers	cross sectional	To assess possible associations between performance level (achieved level of difficulty), chronic injuries and body mass index (BMI) in sport climbing.	Grønhaug et al., 2019 [25]
356 climbing related injuries	NA	528 climbers, aged 29.2 ± 9.5	cross sectional	To investigate the protective mechanisms or risk factors that can be related to the occurrence of hand-climbing-related injuries	Lion et al., 2016 [31]
2292 injuries	NA	2292 unique, unweighted cases	cross sectional	To provide updated national estimates and demographic characteristics of patients presenting to EDs in the US with rock-climbing-associated injuries between 2000 and 2019 and identify age-stratified injury differences by comparing injury characteristics between pediatric and adult patients.	Pirruccio et al., 2022 [27]
2.7/1000 h	acute 61%, Chronic injuries/significant climbing-related pain 39%	52 climbers, aged 7–18	cross sectional	To estimate the injury rate (IR) and to describe injury patterns and mechanisms; to identify injury risk factors in competitive youth	Barrile et al., 2022 [26]

**Table 2:** Quality assessment of included papers (JBI checklist for analytical cross-sectional studies).

Barrile et al., 2022 [26]	Grønhaug et al., 2019 [25]	Pirruccio, et al., 2022 [27]	Woollings, et al., 2015 [30]	Lutter at al., 2019 [29]	Lion, et al., 2016 [31]	
N	Y	Y	Y	Y	Y	Were the criteria for inclusion in the sample clearly defined?
Y	Y	Y	Y	Y	Y	Were the study subjects and the setting described in detail?
Y	Y	Y	Y	Y	Y	Was the exposure measured in a valid and reliable way?
NA	NA	NA	NA	NA	NA	Were objective, standard criteria used for measurement of the condition?
N	N	N	Y	N	N	Were confounding factors identified?
N	N	N	Y	N	N	Were strategies to deal with confounding factors stated?
Y	Y	Y	Y	Y	Y	Were the outcomes measured in a valid and reliable way?
Y	Y	Y	Y	Y	Y	Was appropriate statistical analysis used?
4/8—fair	5/8—fair	5/8—fair	7/8—good	5/8—fair	5/8—fair	total

Y/N—yes/no; NA—not applicable.

## Data Availability

The data presented in this study are available on request from the corresponding author.
